# Pharmacokinetics and pharmacodynamics of locoregional 5 fluorouracil (5FU) in advanced colorectal liver metastases.

**DOI:** 10.1038/bjc.1988.39

**Published:** 1988-02

**Authors:** J. A. Goldberg, D. J. Kerr, N. Willmott, J. H. McKillop, C. S. McArdle

**Affiliations:** University Department of Surgery, Glasgow Royal Infirmary, UK.

## Abstract

By measuring peripheral drug levels in plasma, the effect of combining albumin microspheres with angiotensin II on systemic exposure to 5FU when administered by bolus injection into the hepatic artery in patients with advanced colorectal liver metastases has been assessed. The results suggest that despite hepatic arterial administration of 5FU, there was no reduction in systemic exposure when compared with that associated with intravenous injection of the same dose. Neither albumin microspheres nor angiotensin II appeared to improve the regional advantage. There have been a number of reports relating the plasma levels of cytotoxic agents with pharmacodynamic parameters. We have shown significant direct correlations between 5FU clearance and 1 week post treatment platelet and white cell counts, and an inverse relationship between the area under the 5FU plasma concentration-time curve (AUC) and 1 week post treatment platelet count and white cell count respectively.


*Br. J. Cancer* (1988), **57**, 186–189 © The Macmillan Press Ltd., 1988

### Pharmacokinetics and pharmacodynamics of locoregional 5 fluorouracil (5FU) in advanced colorectal liver metastases

J.A. Goldberg[1], D.J. Kerr[2], N. Willmott[3], J.H. McKillop[4] & C.S. McArdle[1]

[1]*University Department of Surgery, Glasgow Royal Infirmary;* [2]*University Department of Medical Oncology;* [3]*Department of Pharmaceutics, University of Strathclyde; and* [4]*Department of Nuclear Medicine, Glasgow Royal Infirmary, Glasgow, UK.*

**Summary** By measuring peripheral drug levels in plasma, the effect of combining albumin microspheres with angiotensin II on systemic exposure to 5FU when administered by bolus injection into the hepatic artery in patients with advanced colorectal liver metastases has been assessed. The results suggest that despite hepatic arterial administration of 5FU, there was no reduction in systemic exposure when compared with that associated with intravenous injection of the same dose. Neither albumin microspheres nor angiotensin II appeared to improve the regional advantage. There have been a number of reports relating the plasma levels of cytotoxic agents with pharmacodynamic parameters. We have shown significant direct correlations between 5FU clearance and 1 week post treatment platelet and white cell counts, and an inverse relationship between the area under the 5FU plasma concentration-time curve (AUC) and 1 week post treatment platelet count and white cell count respectively.

The failure and toxicity of systemic chemotherapy in the treatment of liver metastases of colorectal origin has led to increasing interest in locoregional therapy (Taylor, 1985). Most antineoplastic agents have steep dose response curves and systemic toxicity can often be related to some aspect of the drug's plasma concentration-time profile (Powis, 1986). The hypothesis underlying rational use of intraarterial chemotherapy is that it should allow the generation of high drug concentrations at the tumour with reduced systemic exposure and hence toxicity (Dedrick *et al.*, 1978). Attempts have been made to increase tumour drug delivery and hence therapeutic index by altering the organ blood flow during cytotoxic drug infusion. Sasaki *et al.* (1985) have shown that infusion of the vasoconstrictor angiotensin II via the hepatic artery increases tumour blood-flow by 50%, relative to the surrounding normal tissue. Ensminger *et al.* (1985) adopted a different approach and demonstrated that the administration of biodegradable starch microspheres (40 $\mu$m diam.) via the hepatic artery, increases the hepatic uptake of mitomycin C, and reduces the concentration of drug in the systemic circulation. Our aim in the present study was to examine the pharmacokinetic and pharmacodynamic advantages of the sequential administration of 5FU with albumin microspheres during angiotensin II infusion via the hepatic artery, in patients with advanced hepatic metastases from colorectal cancer.

### Materials and methods

Nine patients with advanced hepatic metastases from a colorectal primary tumour were included in the study. Estimations of the percentage replacement of liver parenchyma by tumour, were based on Tc99m tin colloid scans performed prior to the study.

All patients underwent insertion of a 'Portacath' arterial silicone catheter and subcutaneous reservoir (I.D. 0.76 mm, O.D. 2.29 mm, system 320, Pharmacia, Pharmacia House, Midsummer Boulevard, Milton Keynes, UK). At laparotomy, the stomach and duodenum were mobilised and the lesser sac opened to identify the gastroduodenal artery at the junction with the hepatic artery. These vessels were mobilised, and a standard cholecystectomy performed to obviate gallbladder ischaemia. The catheter was then placed in a tunnel made between a subcutaneous pocket created on the right chest wall and the peritoneal cavity. The reservoir was then sutured into position over a rib within the subcutaneous pocket. The gastroduodenal artery was ligated distally and cannulated so that the catheter tip lay at the junction of the gastroduodenal and hepatic arteries. The catheter and reservoir were then filled with heparinised saline, which was replaced by flushing daily in the immediate post-operative period, and then once weekly.

Albumin microspheres were prepared by a technique involving stabilisation with glutaraldehyde of a water in oil emulsion containing protein (Lee *et al.*, 1981). The basic system has been modified by us to produce microspheres of a size suitable for entrapment in terminal capillaries. (Willmott *et al.*, 1985). Human serum albumin (600 mg) was dissolved in 1 mM phosphate buffer containing 0.1% SDS (1 ml) and diluted with water (1.8 ml). The albumin solution is emulsified in an oil phase of cotton seed oil and petroleum ether, and the protein cross-linked with 25% gluteraldehyde (150 $\mu$l) to stabilise the microspheres. Following consecutive washes in petroleum ether/isopropanol/PBS/0.5% tween, and PBS/0.2% tween to remove particles $<7\,\mu$m in diameter, the microspheres were ready for use. All procedures were carried out in a sterile products unit which maintains a sterile environment to the required British standard. This ensured that the microspheres were sterile, and an aliquot from each dose was formally checked by solubilisation with trypsin prior to sterility testing. The size of the microspheres was measured by Coulter counter or laser diffraction (Malvern Instruments, Malvern, UK) and was found to be 20–40 $\mu$m in diameter (50% weight average). Between 60 and 90 million particles constitute a bolus dose as used in the pharmacokinetic studies described in this paper.

In a preliminary phase I study to determine the maximum tolerated dose of albumin microspheres, angiotensin II (Ciba Laboratories) was infused at a rate of $10\,\mu g\,min^{-1}$ for 4 min into the hepatic artery. This caused an increase in systolic blood-pressure of between 25 and 40 mm Hg but resulted in no ill effects. A bolus injection of albumin microspheres was given at $t = 100$ sec by the same route followed by a 1 g bolus of 5FU.

Microsphere doses were escalated from 100 to 300 mg in 50 mg increments. Pharmacokinetic studies were performed for each of the four treatments listed below. They were performed at weekly intervals and in random order. For each study, the preparations were administered as immediately consecutive boluses unless otherwise stated.

1. i.v. 5FU: Intravenous injection of 1 g 5FU.
2. i.a. 5FU: Hepatic intraarterial injection of 1 g 5FU.

Correspondence: J.A. Goldberg.
Received 29 June 1987; and in revised form, 20 November 1987.

3. i.a. AMS; 5FU: Hepatic intraarterial injection of 300 mg albumin microspheres (AMS), followed by hepatic intraarterial injection of 1 g 5FU.
4. i.a. AII; AMS; 5FU: Hepatic intraarterial infusion of angiotensin II at a rate of $10\,\mu g\,min^{-1}$ for 4 min (AII); at $t=100$ sec, an hepatic intraarterial injection of AMS, followed by an intrahepatic arterial injection of 1 g 5FU.

Blood samples (10 ml) were withdrawn from a canula in an antecubital vein and collected into lithium heparin tubes before 5-fluorouracil administration, and at intervals thereafter (5, 30, 60, 90, 120, 180 and 240 min). The blood samples were centrifuged (2000 rpm for 5 min) and the plasma separated and stored at −20°C until analysis. 5FU plasma concentrations were measured by a sensitive and specific HPLC method (Christophidis, 1979), with inter- and intra-assay coefficients of variation of between 5 and 10%.

The 5FU plasma concentration values were fitted to a 2-compartment open model by non linear least squares fitting using an in house programme based on the Maquhardt algorithm (Bevington, 1969). AUC was calculated from time zero to infinity by the trapezoidal rule.

The white cell and platelet counts were estimated before and at 1 week after the pharmacokinetic studies. Regression lines were constructed between AUC, total body clearance of 5FU, 5 min serum peak of 5FU, and 1 week post treatment white cell and platelet counts, and the percentage change in white cell and platelet counts following treatment respectively. Base-line tumour arterio-venous shunting was assessed in 5 patients by intraarterial injection of $^{99m}Tc$ labelled albumin microspheres using a glass syringe. The upper abdomen and thorax were scanned, and both anterior and posterior views obtained with a gamma camera (IGE 400 A Tomographic gamma camera with low energy parallel multi-purpose collimator). Regions of interest were drawn around the liver and lungs, and the relative lung uptake (RLU) calculated as RLU = Activity (lung-fields)/Activity (liver + lung-fields).

### Results

Tin colloid scans revealed that the patients studied had extensive replacement of hepatic tissue by tumour (5 patients >50% replacement; 4 patients 25–50% parenchymal replacement). Base-line percent shunting was calculated as 1.41 ± 0.4% (mean ± s.d.), and ranged from 0.4–2.9%.

The maximum dose of albumin microspheres administered via the hepatic artery was 350 mg. This dose caused significant right upper quadrant pain in 4 out of 5 patients studied, so that the recommended dose for clinical trials, and that used in these pharmacokinetic studies was 300 mg. The pharmacokinetic results are summarised in Table I.

The plasma 5FU concentrations fitted a 2 compartment model in most cases, and it was possible to derive drug clearance and volume of distribution from the model parameters. It is clear from Table I and Figure 1 that the pharmacokinetics of intraarterial 5FU do not differ markedly from those following bolus intravenous administration.

**Figure 1** Plasma 5FU concentration – time curves for each bolus treatment (mean ± s.d.), (a) Intravenous injection, 1 g 5FU; (b) Hepatic arterial injection, 1 g 5FU; (c) Hepatic arterial injection, AMS; 1 g 5FU; (d) Hepatic arterial injection of AII; AMS; 1 g 5FU.

With regard to the pharmacodynamics of 5FU, there were significant linear correlations between plasma clearance and

**Table I** Pharmacokinetic studies of intravenous and intrahepatic arterial bolus injection of 5FU (with and without albumin microspheres and angiotensin II) in patients with advanced colorectal liver metastases

| | *n* | *AUC* *mg l min⁻¹* | *Clearance* *l min⁻¹* | *5 min* *peak mg l⁻¹* | $t_{1/2}$ *min* |
|---|---|---|---|---|---|
| 1. i.v. 5FU | 9 | 1172 ± 365 | 0.94 ± 0.3 | 39 ± 11 | 17 ± 5 |
| 2. i.a. 5FU. | 9 | 1312 ± 325 | 0.81 ± 0.2 | 43 ± 10 | 17 ± 6 |
| 3. i.a. AMS; 5FU | 6 | 1115 ± 481 | 1.01 ± 0.3 | 35 ± 7 | 17 ± 6 |
| 4. i.a. AII; AMS; 5FU | 5 | 1403 ± 461 | 0.78 ± 0.3 | 39 ± 9 | 17 ± 3 |

AUC = Area under the plasma 5FU concentration – time curve; $t_{1/2}$ = terminal disposition phase half-life of 5FU in plasma; All values expressed as mean ± s.d.

1-week post treatment white-cell count ($r=0.69$; $P=0.48$) and platelets ($r=0.89$; $P=0.8$). There are significant inverse linear correlations between AUC for 5FU and 1-week post treatment white cell and platelet counts ($r=0.66$, $P=0.44$; $r=0.88$, $P=0.77$ respectively) (Figure 2).

Of the 9 patients included in the study, 2 died 6.6 and 7.2 months respectively after catheter implantation from disease progression. The 7 patients still living have been receiving locoregional 5 fluorouracil therapy for $4.3 \pm 2.6$ months (mean $\pm$ s.d.) at the time of writing. One patient has had disease progression and was withdrawn from the programme 8.5 months after catheter implantation. The remaining 6 patients currently have stable disease as assessed by ultrasound and tin colloid scan.

### Discussion

In this study, we have attempted to manipulate the pharmacokinetics of 5FU so as to maximise tumour exposure and decrease drug delivery to the systemic compartment. Hepatic 5FU extraction has been measured in patients with metastatic cancer to the liver and has been found to be as high as 50% on first pass (Ensminger et al., 1978). If 50% of 5FU were extracted on first pass following intraarterial bolus administration as in the present study, then by using a theoretical model, one could predict that drug levels reaching the systemic circulation would be one half of those following intravenous administration of the same dose. Measures taken to increase tumour drug delivery by temporarily altering blood flow with vasoactive agents, and causing a transient reduction in hepatic blood flow by embolising the hepatic microvascular bed with degradable albumin microspheres should help to increase hepatic drug extraction and decrease systemic exposure.

**Figure 2** The correlation between the area under the plasma 5FU concentration – time curve and the platelet count 1 week after injection of 5FU. ($r=0.876$; $P=0.767$).

No differences in the pharmacokinetics of 5FU were seen when standard systemic was compared with administration via the hepatic artery. There are a number of possible explanations for these findings.

Although we have described the kinetics of 5FU using a linear model, there is evidence to suggest that the drug has non linear pharmacokinetics after hepaticarterial and intravenous infusions in cancer patients (Collins et al., 1980; Wagner et al., 1986).

If there is a saturable mechanism for hepatic extraction of 5FU, then delivery of an intraarterial bolus with generation of high drug concentrations within the hepatic vascular compartment could exceed the capacity for tissue uptake and allow a larger than expected drug dose to reach the systemic circulation. Reduction of the 5FU infusion rate could therefore increase the hepatic extraction and reduce systemic exposure to the cytotoxic agent.

The patients reported in this study had a large hepatic tumour burden and there is some evidence to suggest that patients with advanced liver tumours have a reduced hepatic extraction capacity (Heidelberger, 1975; Mukherjee et al., 1963). There are supportive animal experimental data for this hypothesis as rats with liver tumours have been shown to have an insignificant capacity for metabolic degradation of pyrimidines (Weber et al., 1971).

A further contributory factor to the apparent loss of regional advantage through administering 5FU via the hepatic artery may be the existence of arterio-venous shunts. However, we found negligible base line shunting in 5 patients studied (Goldberg et al., 1987), so that this would not account for the similarity of results between intravenous and intrahepatic arterial 5FU.

Further studies are planned on patients with early metastatic disease, and we intend to assess the effects of slower infusion rates of 5FU, and varying the order of microsphere administration in an attempt to enhance regional selectivity.

The relationship between the pharmacokinetics and pharmacodynamics of anti-cancer drugs has recently been reviewed by Powis (1986). There are a number of reports relating peak plasma levels of 5FU and its AUC, with regard to 5FU and gastrointestinal toxicity (Au et al., 1982; Byfield et al., 1983). In the present study significant correlations between 5FU clearance and 1-week post treatment platelet and white cell counts, and inverse correlations between AUC and 1-week post treatment platelet and white cell counts have been demonstrated. This implies that any attempt to reduce the systemic 5FU exposure, as manifest by the AUC, and increase tumour exposure and drug clearance, should result in decreased myelotoxicity.

The Authors gratefully acknowledge the financial support of the Cancer Research Campaign and Medical Research Council, thank Messers A. Setanoiars and M. Bradnam for their expert technical assistance, and are indebted to Ciba Laboratories for the provision of angiotensin II.

### References

AU, J.L.S., RUSTUM, Y.M., LEDESMA, E.J., MITTLEMAN, A. & CREAVEN, P.J. (1982). Clinical pharmacological studies of concurrent infusion of 5-fluorouracil and thymidine in treatment of colorectal carcinomas. *Cancer Res.*, **42,** 2930.

BEVINGTON, P.R. (1969). *Data reduction and error analysis for the physical sciences.* McGraw-Hill: New York, p. 235.

BYFIELD, J.E., FRANKEL, S.S., HORNBECK, C.L., SHARP, T.R. & FLOYD, R.A. (1983). Relationships between serum 5FU level (5FU) and toxicity. *Proc. Am. Soc. Clin. Oncol.*, **2,** 44.

CHRISTOPHIDIS, N., MIHALY, G. VAJDA, F. & LOUIS, W. (1979). Comparison of gas and gas-liquid chromatographic assays of 5-Fluorouracil in plasma. *Clin. Chem.*, **25,** 83.

COLLINS, J., DEDRICK, R.L., KING, F.G., SPEYER, J.L. & MYERS, C.E. (1985). Nonlinear pharmacokinetic models for 5-fluorouracil in man: Intravenous and intraperitoneal routes. *Clin. Pharmacol. Ther.*, **28**(2), 235.

DEDRICK, R.L., MYERS, C.E., BUNGAY, P.M. & DE VITA, J.R. (1978). Pharmacokinetic rational for peritoneal drug administration in the treatment of ovarian cancer. *Cancer Treat. Rep.*, **62,** 1.

ENSMINGER, W.D., GYVES, J.W., STEVSON, P. & WALKER-ANDREWS, S. (1985). Phase I study of hepatic arterial degradable starch microspheres and mitomycin C. *Cancer Res.*, **45,** 4464.

ENSMINGER, W.D., ROSOWSKI, A., RASO, V. & 5 others (1978). A clinical-pharmacological evaluation of hepatic arterial infusions of 5-Fluoro-2-deoxyuridine and 5-Fluorouracil. *Cancer Res.*, **38**, 3784.

GOLDBERG, J.A., BRADNAM, M.S., KERR, D.J. & 5 others (1987). Arterio-venous shunting of microspheres in patients with colorectal liver metastases: Errors in assessment due to free pertechnetate and the effect of Angiotensin II. *Nuc. Med. Comm.* (in press).

HEIDELBERGER, C. (1975). Fluorinated pyrimidines and their nucleotides. In *Antineoplastic and immunosuppressive agents*. Part II. Sartorelli and Johns, (eds) p. 193. Springer-Verlag: New York.

LEE, T.K., SOKOLSKI, J.D. & ROYER, G.P. (1981). Serum albumin beads: An injectible, biodegradable system for the sustained release of drugs. *Science*, **213**, 233.

MUKHERJEE, K., BOOHAR, J., WENTLAND, D., ANSFIELD, F.J. & HEIDELBERGER, C. (1963). Studies on fluorinated pyrimidines. XVI. Metabolism of 5-fluorouracil-2-$C^{14}$. *Cancer Res.*, **23**, 49.

POWIS, G. (1986). Anticancer drug pharmacodynamics. *Cancer Chemother. Pharmacol.*, **14**(3), 177.

SASAKI, Y., IMAOKA, S., HASEGAWA, Y. & 7 others (1985). Changes in distribution of hepatic blood flow induced by intra-arterial infusion of angiotensin II in human hepatic cancer. *Cancer*, **55**, 311.

TAYLOR, I. (1985). Colorectal liver metastases – to treat or not to treat? *Br. J. Surg.*, **72**, 511.

WAGNER, J.G., GYVES, J.W., STETSON, P.L. & 4 others (1986). Steady-state nonlinear pharmacokinetics of 5-Fluorouracil during hepatic arterial and intravenous infusions in cancer patients. *Cancer Res.*, **46**, 1499.

WEBER, G., QUEENER, S.F. & FERDINANDUS, J.A. (1971). Control of gene expression in carbohydrate, pyrimidine and DNA metabolism. *Adv. Enzyme Regul.*, **9**, 63.

WILLMOTT, N., CUMMINGS, J., STUART, J.F.B. & FLORENCE, A.T. (1985). Adriamycin loaded albumin microspheres: Preparation, *in-vivo* distribution and release in the rat. *Biopharmacol. Drug Dispos.*, **6**, 91.


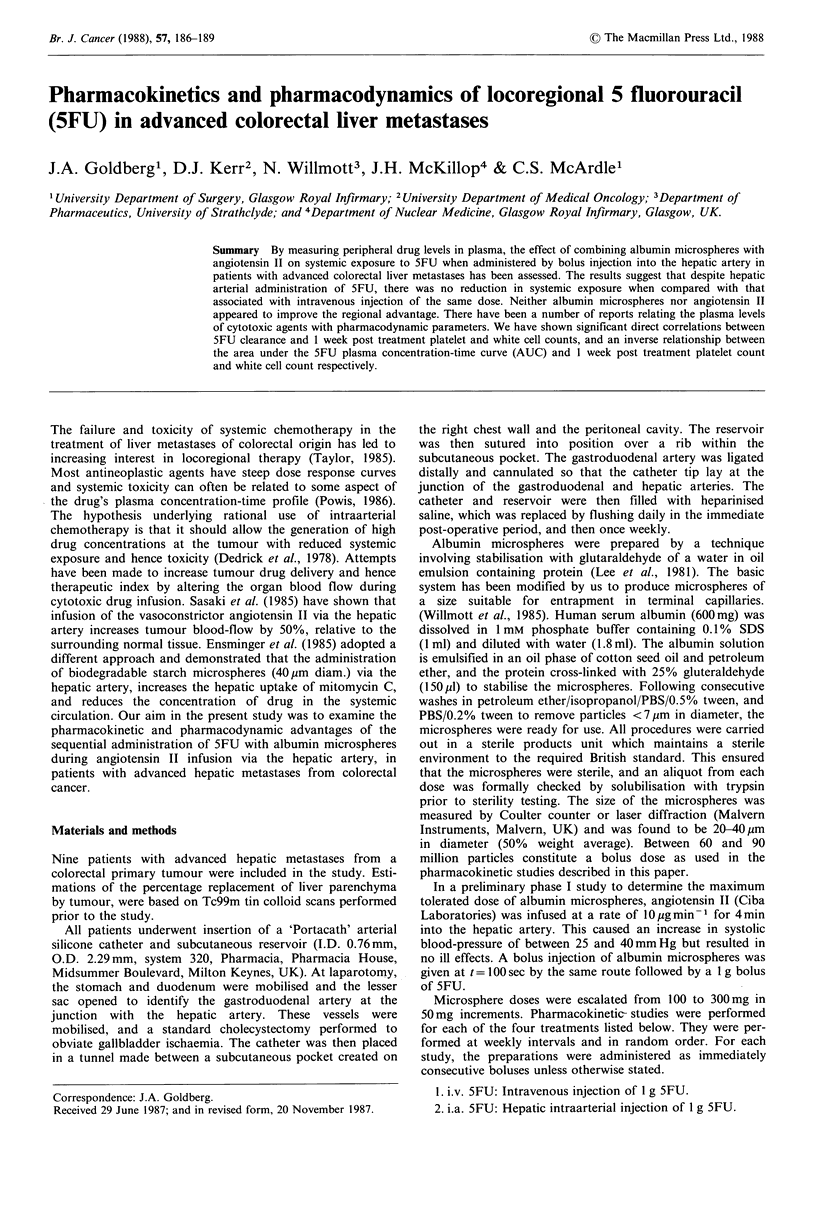

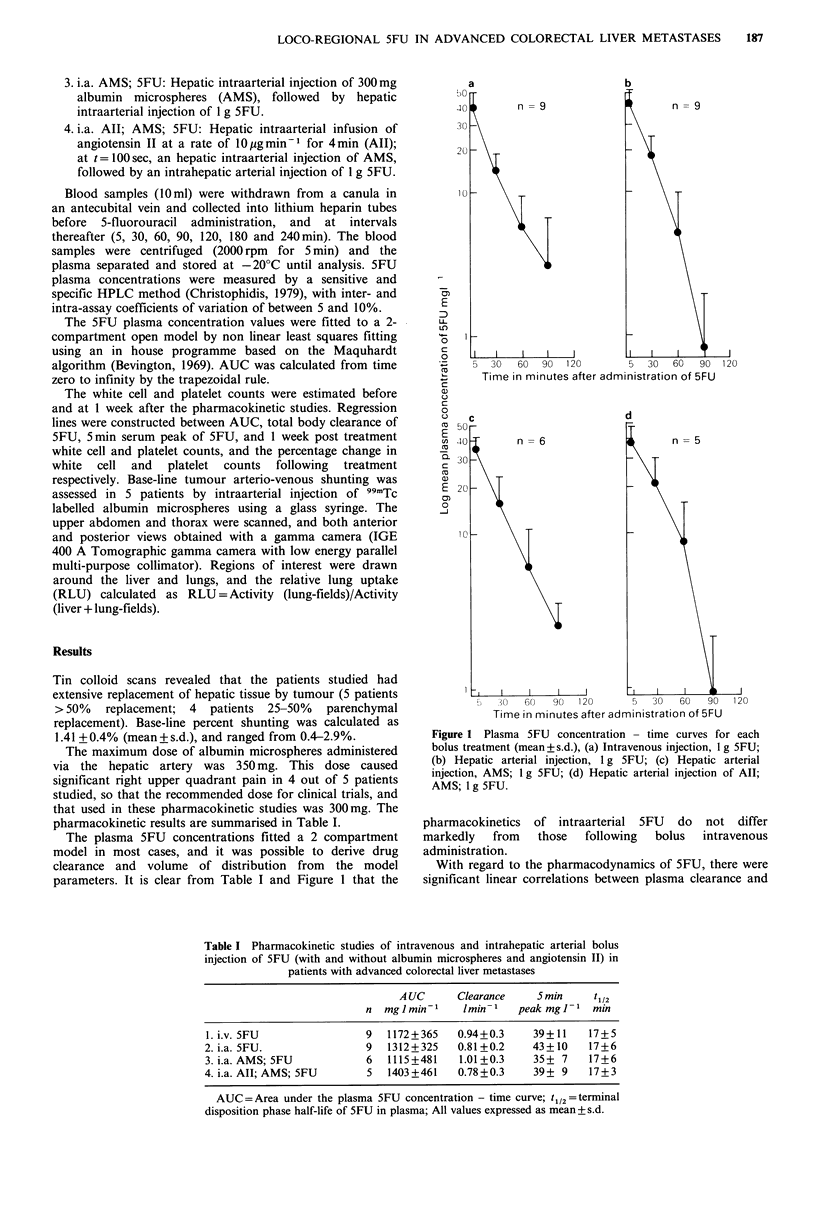

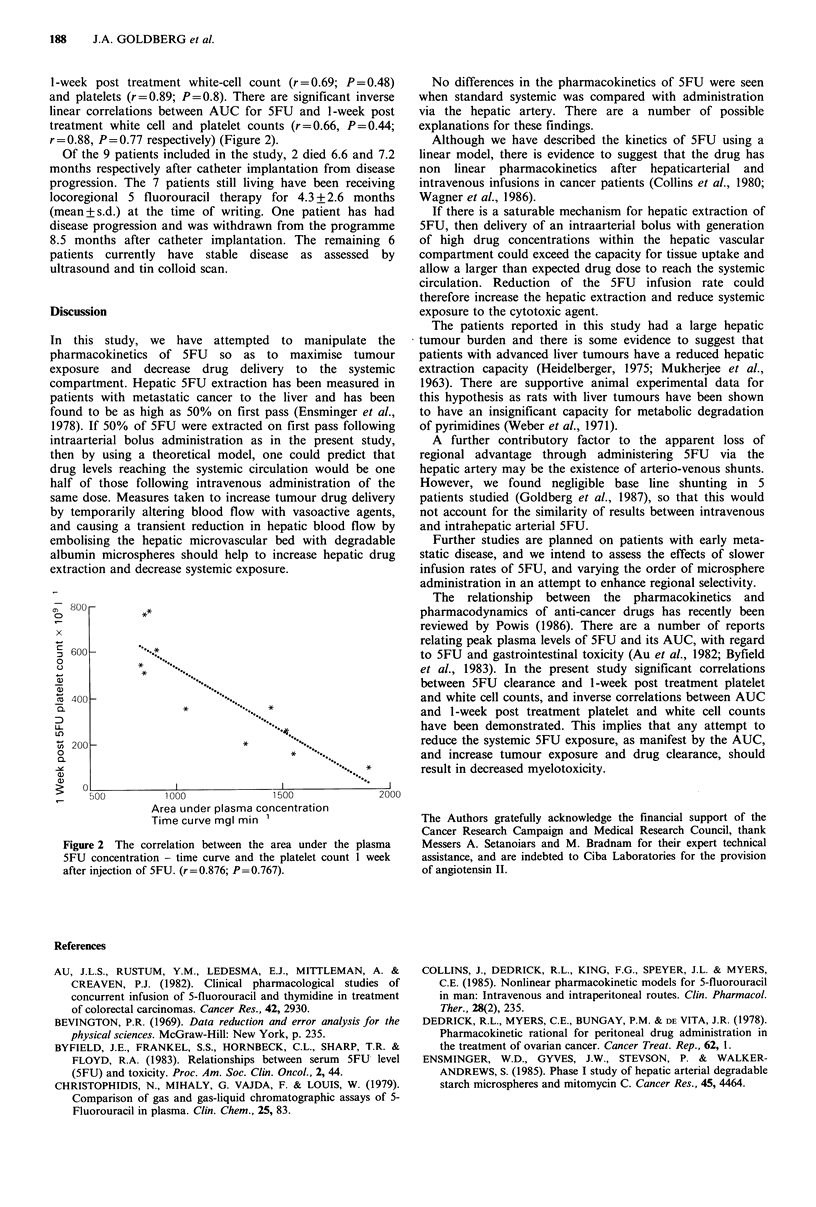

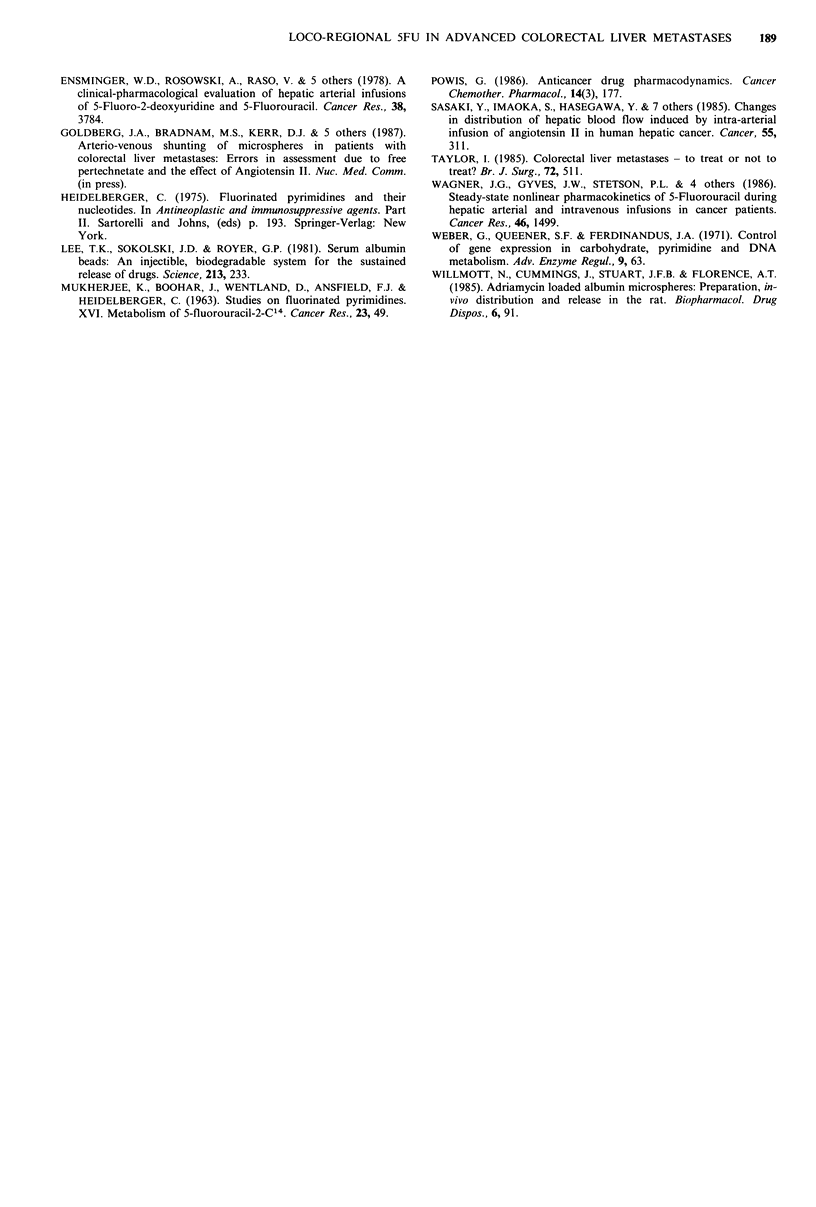


## References

[OCR_00407] Au J. L., Rustum Y. M., Ledesma E. J., Mittelman A., Creaven P. J. (1982). Clinical pharmacological studies of concurrent infusion of 5-fluorouracil and thymidine in treatment of colorectal carcinomas.. Cancer Res.

[OCR_00427] Collins J. M., Dedrick R. L., King F. G., Speyer J. L., Myers C. E. (1980). Nonlinear pharmacokinetic models for 5-fluorouracil in man: intravenous and intraperitoneal routes.. Clin Pharmacol Ther.

[OCR_00433] Dedrick R. L., Myers C. E., Bungay P. M., DeVita V. T. (1978). Pharmacokinetic rationale for peritoneal drug administration in the treatment of ovarian cancer.. Cancer Treat Rep.

[OCR_00440] Ensminger W. D., Gyves J. W., Stetson P., Walker-Andrews S. (1985). Phase I study of hepatic arterial degradable starch microspheres and mitomycin.. Cancer Res.

[OCR_00445] Ensminger W. D., Rosowsky A., Raso V., Levin D. C., Glode M., Come S., Steele G., Frei E. (1978). A clinical-pharmacological evaluation of hepatic arterial infusions of 5-fluoro-2'-deoxyuridine and 5-fluorouracil.. Cancer Res.

[OCR_00464] Lee T. K., Sokoloski T. D., Royer G. P. (1981). Serum albumin beads: an injectable, biodegradable system for the sustained release of drugs.. Science.

[OCR_00474] Powis G. (1985). Anticancer drug pharmacodynamics.. Cancer Chemother Pharmacol.

[OCR_00478] Sasaki Y., Imaoka S., Hasegawa Y., Nakano S., Ishikawa O., Ohigashi H., Taniguchi K., Koyama H., Iwanaga T., Terasawa T. (1985). Changes in distribution of hepatic blood flow induced by intra-arterial infusion of angiotensin II in human hepatic cancer.. Cancer.

[OCR_00484] Taylor I. (1985). Colorectal liver metastases--to treat or not to treat?. Br J Surg.

[OCR_00488] Wagner J. G., Gyves J. W., Stetson P. L., Walker-Andrews S. C., Wollner I. S., Cochran M. K., Ensminger W. D. (1986). Steady-state nonlinear pharmacokinetics of 5-fluorouracil during hepatic arterial and intravenous infusions in cancer patients.. Cancer Res.

[OCR_00494] Weber G., Queener S. F., Ferdinandus J. A. (1970). Control of gene expression in carbohydrate, pyrimidine and DNA metabolism.. Adv Enzyme Regul.

[OCR_00499] Willmott N., Cummings J., Stuart J. F., Florence A. T. (1985). Adriamycin-loaded albumin microspheres: preparation, in vivo distribution and release in the rat.. Biopharm Drug Dispos.

